# Infection control measures in response to detection of carbapenem-resistant enterobacterales in neonatal intensive care unit

**DOI:** 10.1017/ash.2025.126

**Published:** 2025-09-03

**Authors:** Hee Jeong Wang, Ji Yeoung Yim, Na Yoon Kim, Go Eun Kim, Ji Eun Park, Yun Ah Choi, Seo Young Yoo, Young Hwa Choi, Hyun Joo Jung

**Affiliations:** 1 Infection Control OfficeAjou University Hospital, Suwon, Korea; 2 Department of Infectious DiseasesAjou University Hospital, Suwon, Korea; 3 Department of Pediatrics and AdolescentsAjou University Hospital, Suwon, Korea

**Keywords:** Carbapenem-resistant Enterobacterales, Neonatal intensive care unit

## Abstract

**Objectives:** Neonatal intensive care unit (NICU) admits premature babies and neonates with acute illness who are under high infection risk due to immature immune response system. Infections caused by carbapenem- resistant Enterobacterales (CRE) is a serious threat to such patient population. A single case of CRE infection occurred in 36-bed NICU on July 2023. Infection control measures were put in place to prevent further CRE infection within the NICU. **Methods:** A neonate delivered at gestational age of 23 weeks and 6 days with birth weight of 650g was under mechanical ventilator care. On 35^th^ day of life, CRE (*Escherichia coli,* New Delhi metallo-beta- lactamase-1 positive) was isolated from this neonate’s endotracheal suction material. After discussion with infection control physician, the bacterial culture was determined to have been resulted from colonization or localized infection, rather than invasive infection. Five measures were taken to prevent additional infection within the NICU. One, contact precaution was issued for CRE-infected baby, and an isolation ward and a designated nurse was assigned for the baby to prevent cross infection. Two, adherence to hand hygiene and personal protective equipment (PPE) application was monitored for medical personnel and visitors entering the NICU. Three, a checklist was designed specifically for disinfection of NICU isolation ward, and the designated cleaner and assistants were educated on the checklist. Four, testing with fluorescent markers was performed to validate cleaning. Five, the infectious disease specialist and the pharmacy analyzed the prescription pattern of broad-spectrum antibiotic among patients in NICU for systematic antibiotic regulation. **Results:** The following results were obtained after 2 weeks of infection control measures. 57 subjects underwent hand hygiene monitoring, on which 15.8%(9 case) of the subjects unadequately passed. Immediate feedback was provided upon these detections. Cleaning validation detected a single cases of inadequate disinfection (door to isolation ward), for which re-cleaning and education was performed. An increasing trend in consumption of 3^rd^ generation cephalosporin (8.96% in April 2023 to 21.21% in June 2023) was found, and the neonatology department was advised to be more selective in prescription of broad- spectrum antibiotics. **Conclusions:** There was no CRE infection for 6 months following infection control measures. This case was determined to be an isolated case of CRE infection, and no further surveillance culture was obtained. Proactive infection control measures, including contact isolation, hand hygiene, environment cleaning, and regulation of broad-spectrum antibiotics, are necessary to prevent secondary infections that may follow an index CRE infection in the NICU.

